# Supplementary material for: The neurodevelopmental transcriptome of the Drosophila melanogaster microcephaly gene abnormal spindle reveals a role for temporal transcription factors and the immune system in regulating brain size
Source: bioRxiv. 2023 Jan 10:2023.01.09.523369. Preprint. [Version 1] doi: 10.1101/2023.01.09.523369 (PMC9882087; doi:10.1101/2023.01.09.523369)
Supplement: Supplement 1 — Supplementary Figure 1, Related to Figure 1. Brain size measurements and mitotic spindle morphology. (A) Cartoon of the larval and pupal/adult brain. The optic lobes of the larval brain consists of two neuroblast (NBs) regions that will give rise to distinct neuron and glial populations in the adult brain. Central brain NBs (green) make neurons and glia for the central brain of the adult, optic lobe NBs (also known as medulla neuroblasts) and neuroepithelial cells (red) generate the neurons and glia of the adult optic lobe. For μ-CT measurements, the ‘entire brain’ consists of both larval optic lobes (blue outline) segmented as a whole, while the pupal and adult entire brain consists of both optic lobe regions plus the central brain. ‘Optic lobe’ measurements were taken from individually segmented optic lobes of the pupa and adult. The larval ventral nerve cord was not included in the size analysis, although it was included in the flow cytometry analysis due to the difficulty in accurately separating it from the optic lobes during dissection. μ-CT measurements of wildtype (WT, aspT25/+) and asp mutant (aspT25/aspDf) volume from (B) larva entire brain, (C) pupa entire brain, (D) pupa optic lobe, (E) adult entire brain, (F) adult optic lobe. μ-CT measurements of asp rescue control (ubi-GFP::aspMF/+; aspT25/+) and asp rescue (ubi-GFP::aspMF/+; aspT25/aspDf) volume from (G) larval entire brain, (H) adult entire brain, and (I) adult optic lobes. Data is represented as the T-ratio (brain volume normalized to overall body size) and each dot represents a single brain. These values were used to generate the ‘normalized’ graphs in Figure 1B, 1C. For violin plots, solid red line represents the median, and the dashed lines denote interquartile range (IQR). (J) Metaphase mitotic spindles of larval neuroepithelial cells undergoing symmetric division, labeled with anti-β-tubulin to visualize microtubules and anti-pH3 to visualize mitotic chromosomes in WT (aspT25/+), asp mutant (a [file NIHPP2023.01.09.523369v1-supplement-1.pdf]

bioRxiv preprint doi: https://doi.org/10.1101/2023.01.09.523369; this version posted January 10, 2023. The copyright holder for this preprint (which was not certified by peer review) is the author/funder, who has granted bioRxiv a license to display the preprint in perpetuity. It is made available under aCC-BY 4.0 International license.

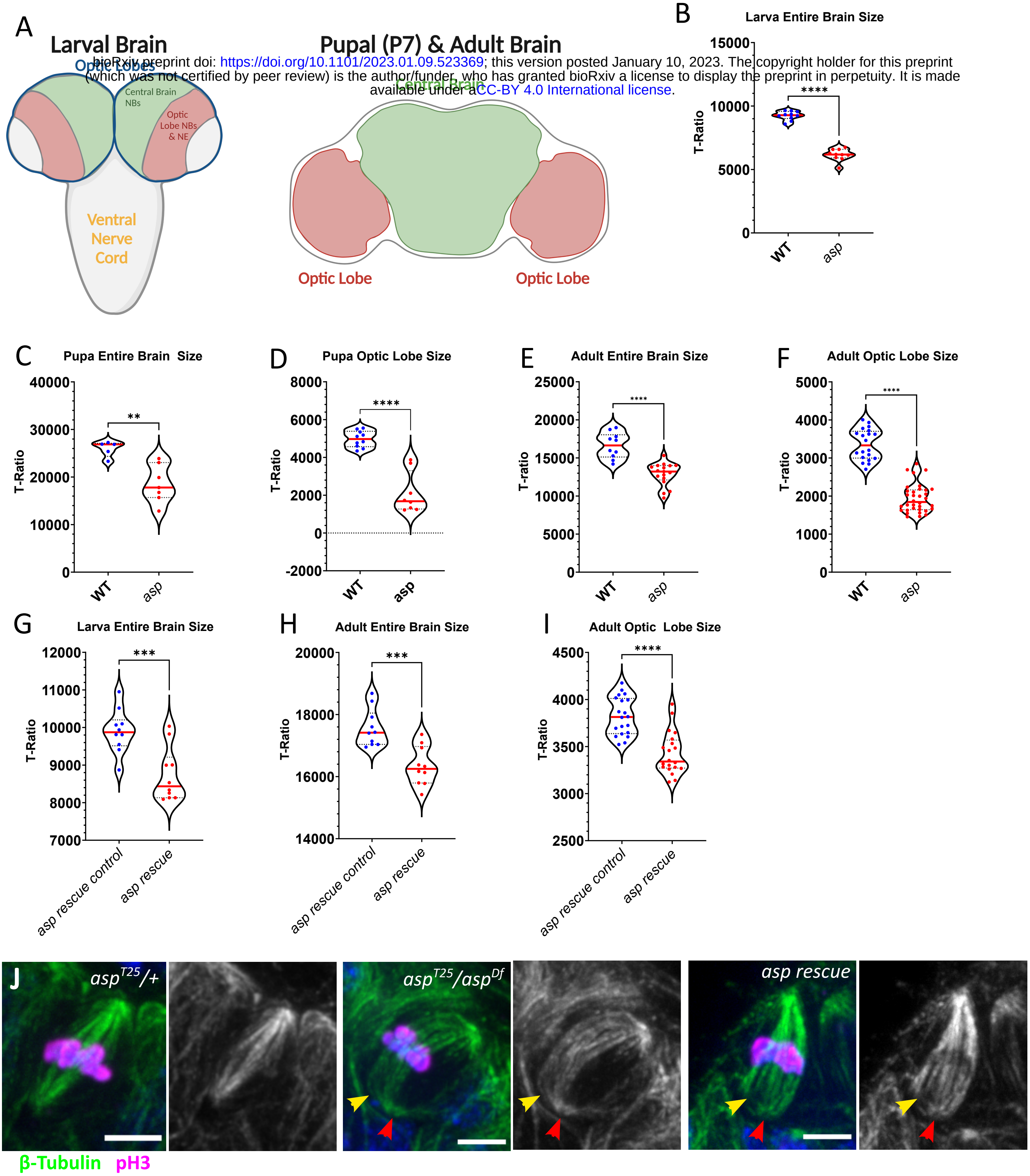

Mannino Supplementary Figure 1

bioRxiv preprint doi: https://doi.org/10.1101/2023.01.09.523369; this version posted January 10, 2023. The copyright holder for this preprint (which was not certified by peer review) is the author/funder, who has granted bioRxiv a license to display the preprint in perpetuity. It is made available under aCC-BY 4.0 International license.

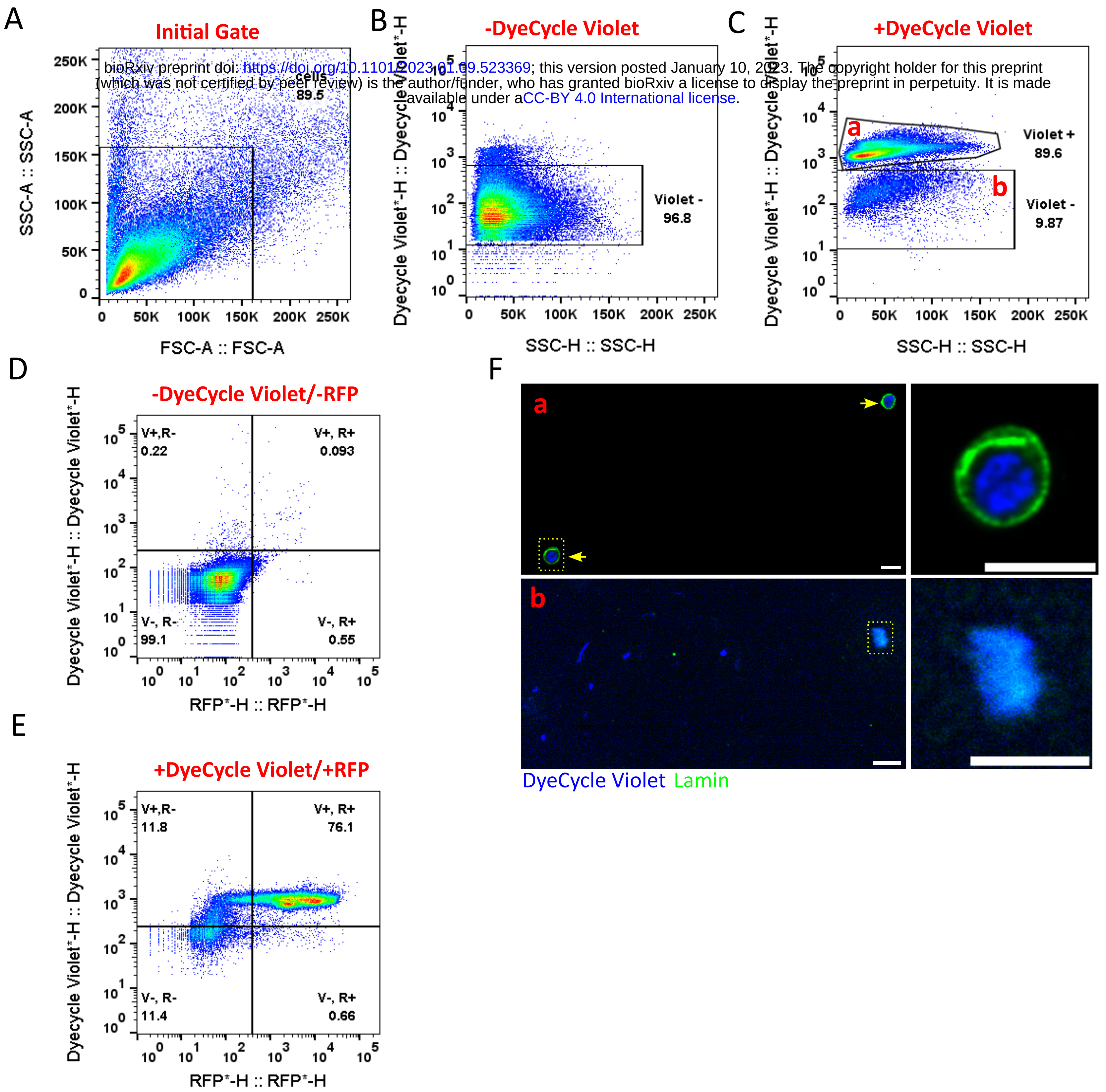

Mannino Supplementary Figure 2

bioRxiv preprint doi: https://doi.org/10.1101/2023.01.09.523369; this version posted January 10, 2023. The copyright holder for this preprint (which was not certified by peer review) is the author/funder, who has granted bioRxiv a license to display the preprint in perpetuity. It is made available under aCC-BY 4.0 International license.

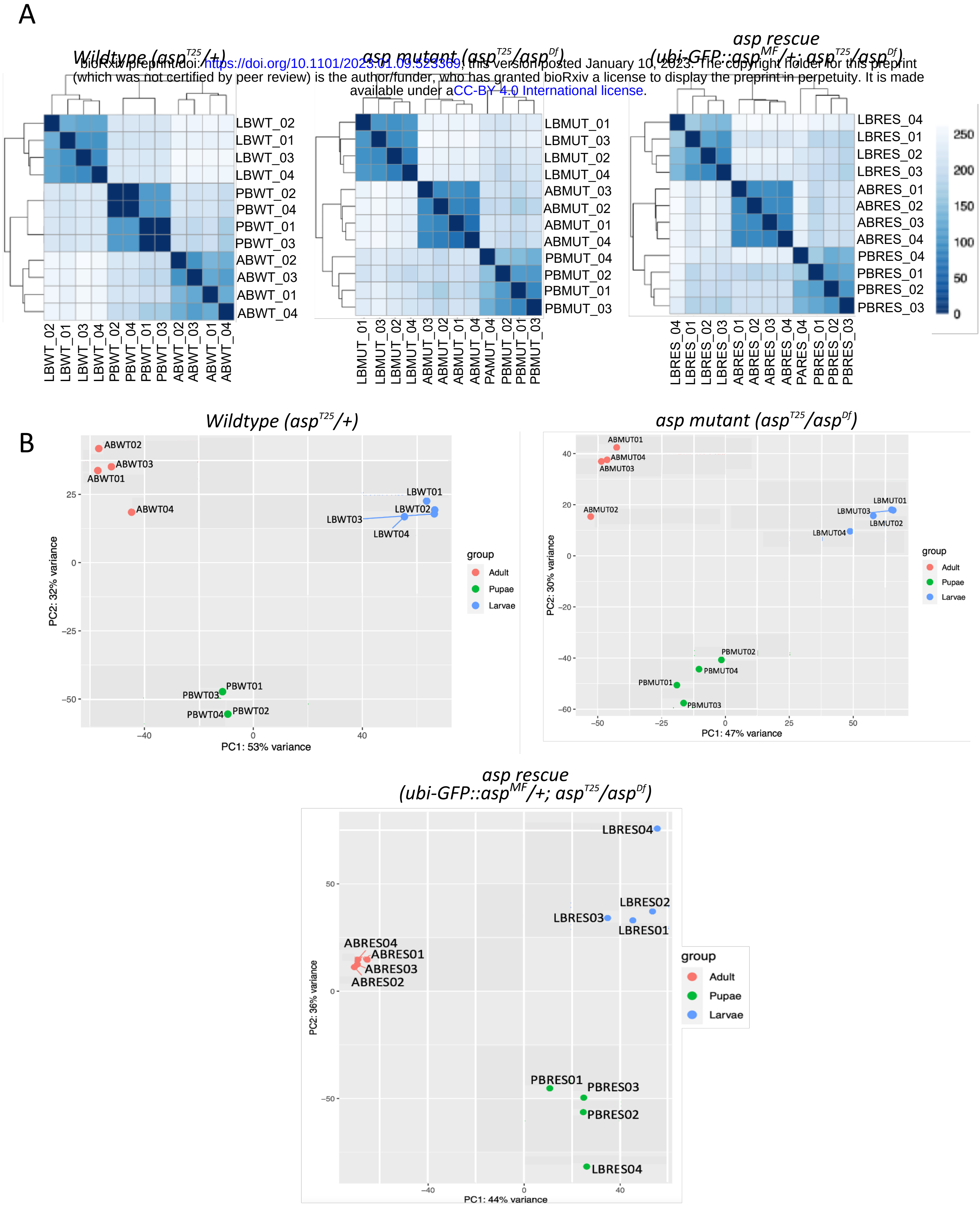

Mannino Supplementary Figure 3

bioRxiv preprint doi: https://doi.org/10.1101/2023.01.09.523369; this version posted January 10, 2023. The copyright holder for this preprint (which was not certified by peer review) is the author/funder, who has granted bioRxiv a license to display the preprint in perpetuity. It is made available under aCC-BY 4.0 International license.

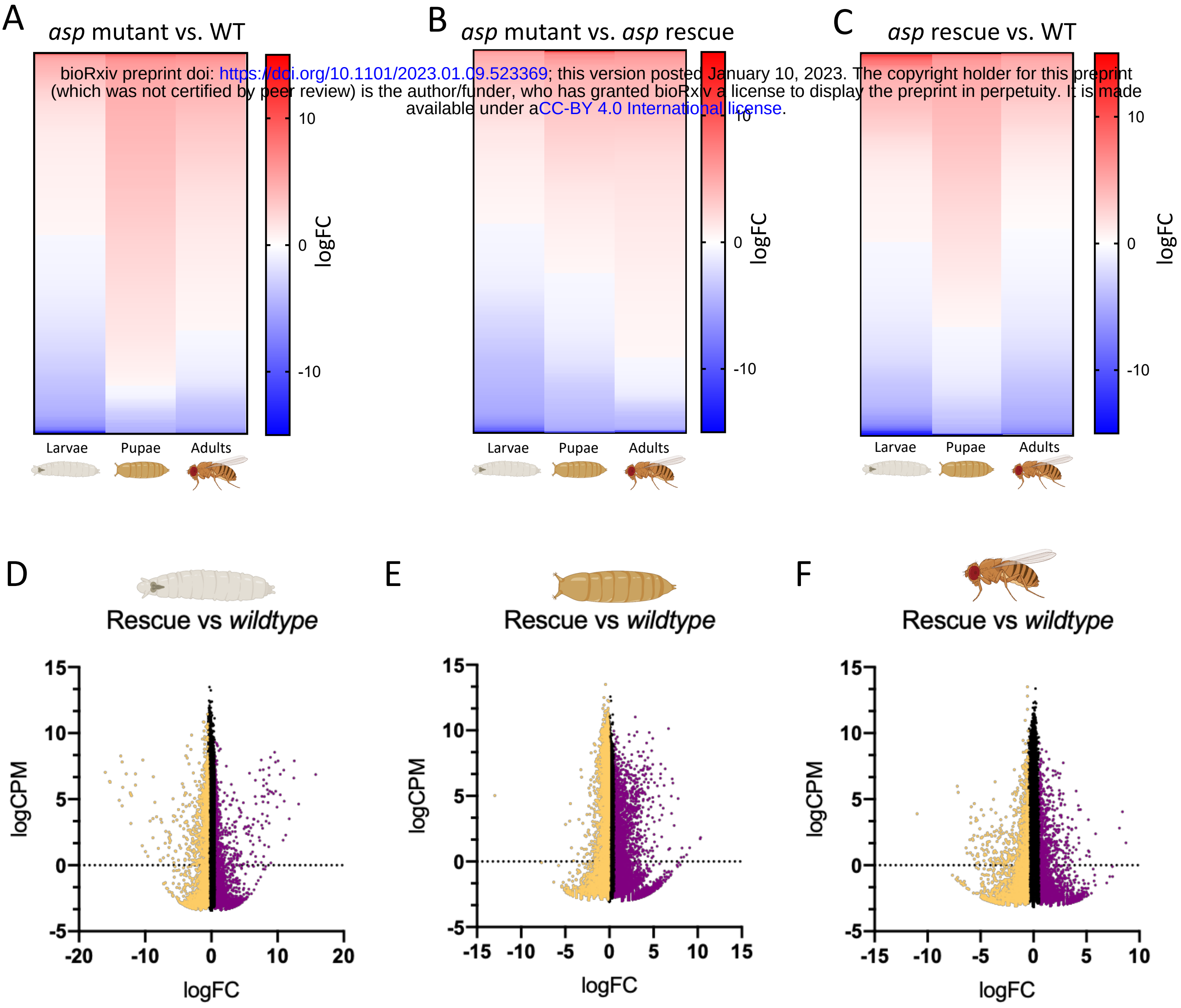

Mannino Supplementary Figure 4

bioRxiv preprint doi: https://doi.org/10.1101/2023.01.09.523369; this version posted January 10, 2023. The copyright holder for this preprint (which was not certified by peer review) is the author/funder, who has granted bioRxiv a license to display the preprint in perpetuity. It is made available under aCC-BY 4.0 International license.

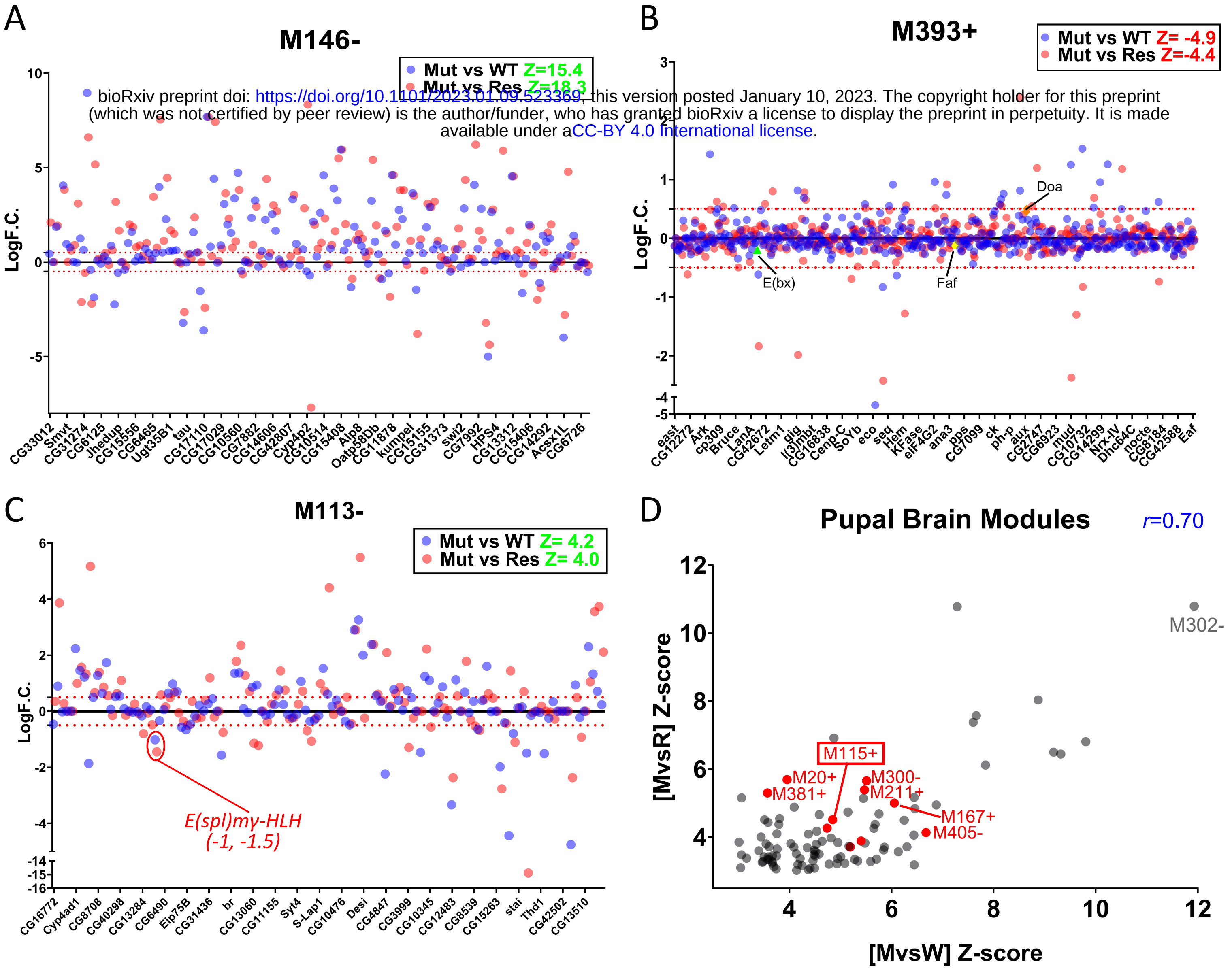

Mannino Supplementary Figure 5

bioRxiv preprint doi: https://doi.org/10.1101/2023.01.09.523369; this version posted January 10, 2023. The copyright holder for this preprint (which was not certified by peer review) is the author/funder, who has granted bioRxiv a license to display the preprint in perpetuity. It is made available under aCC-BY 4.0 International license.

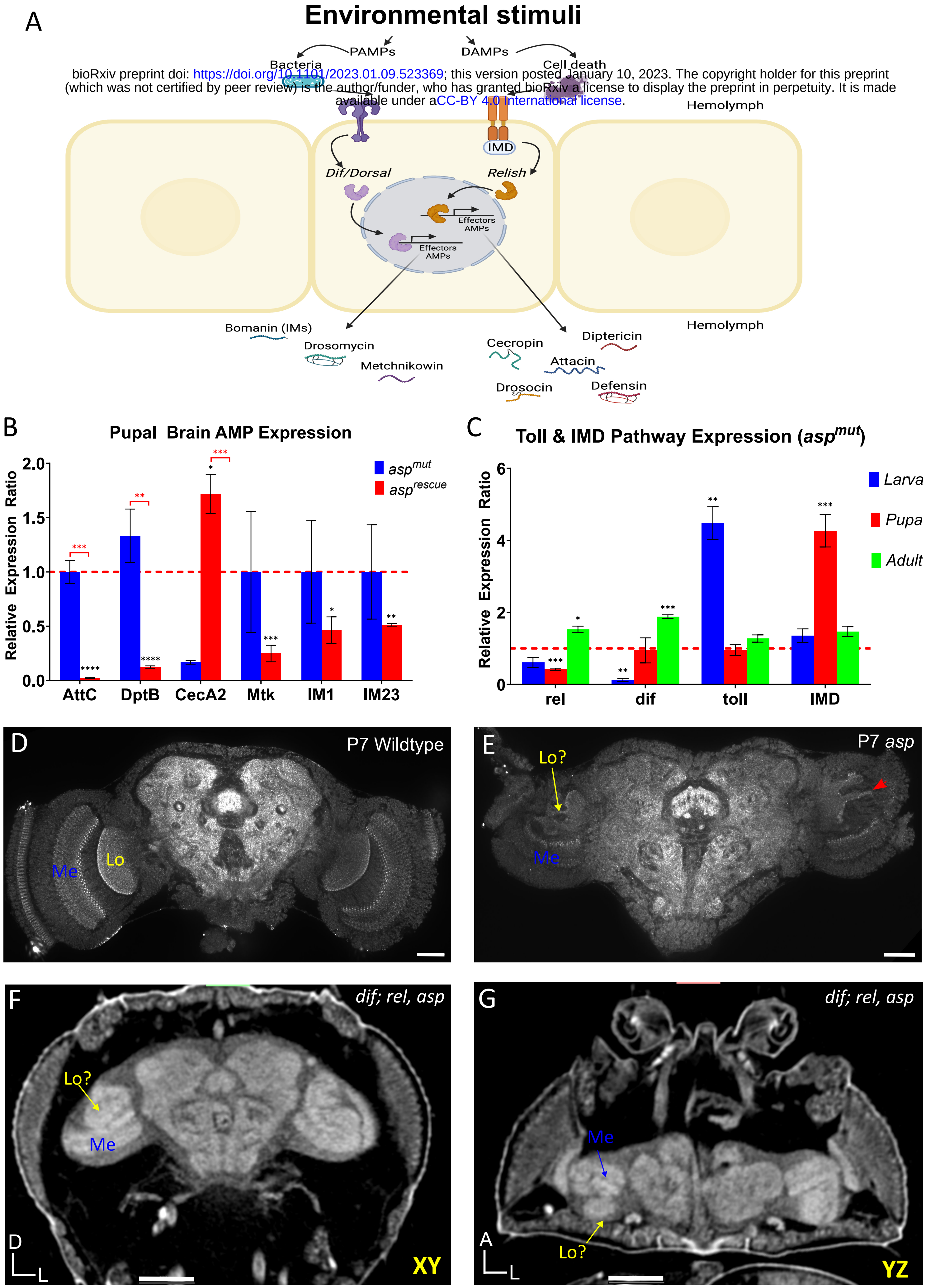

Mannino Supplementary Figure 6

bioRxiv preprint doi: https://doi.org/10.1101/2023.01.09.523369; this version posted January 10, 2023. The copyright holder for this preprint (which was not certified by peer review) is the author/funder, who has granted bioRxiv a license to display the preprint in perpetuity. It is made available under aCC-BY 4.0 International license.

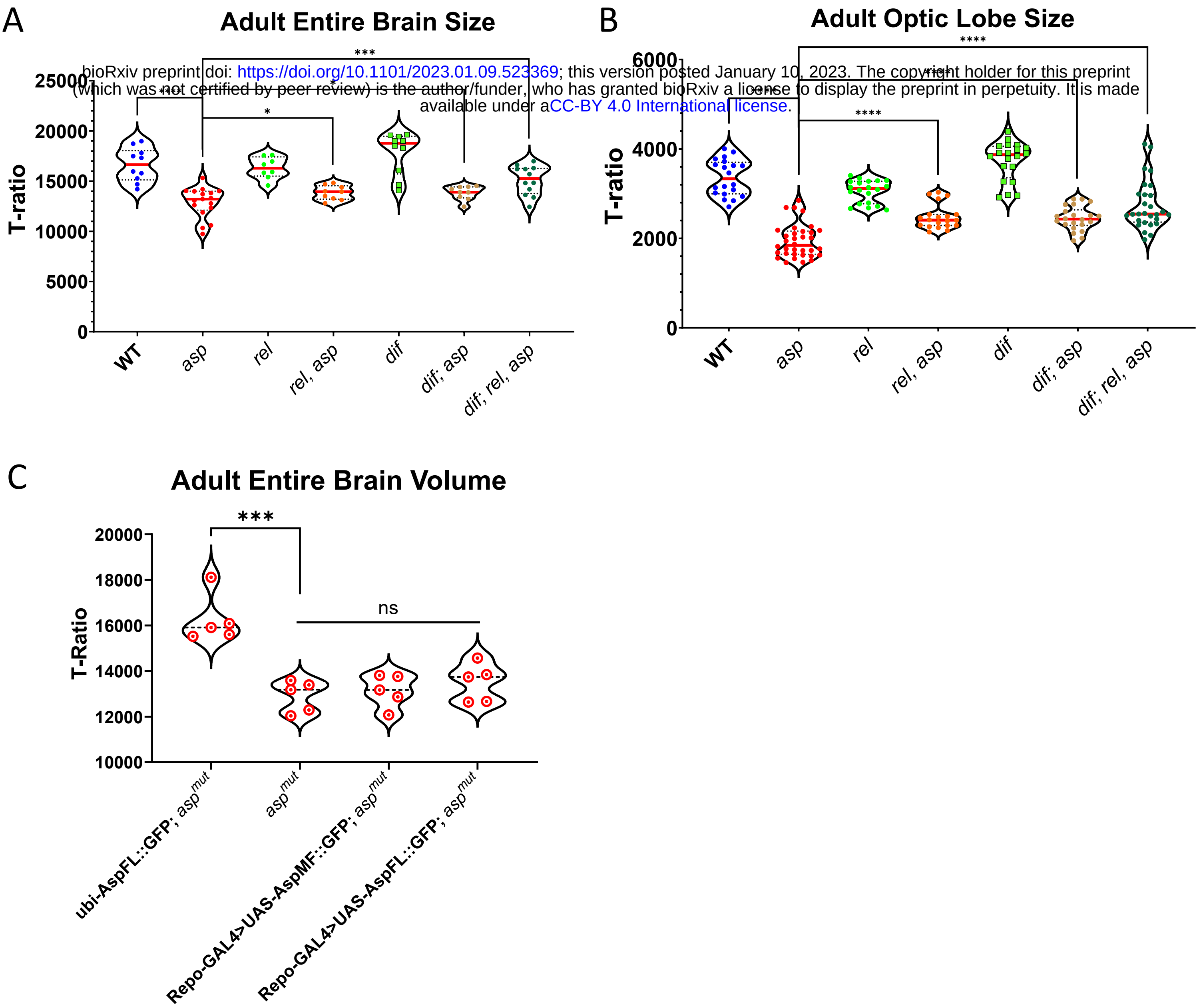

Mannino Supplementary Figure 7
